# Psychosocial determinants of exercise behavior in Parkinson’s disease: a mini-review

**DOI:** 10.3389/fpubh.2026.1942675

**Published:** 2026-09-03

**Authors:** Guanyu Zhang

**Affiliations:** China Institute of Sport Science, Beijing, China

**Keywords:** exercise behavior, Parkinson’s disease, psychosocial determinants, self-efficacy, social support

## Abstract

Parkinson’s disease (PD) is the second most common neurodegenerative disorder, characterized by progressive motor and non-motor symptoms that profoundly impact quality of life. Physical exercise has emerged as a promising non-pharmacological intervention with neuroprotective potential, yet adherence rates remain suboptimal. This mini-review synthesizes current evidence on psychosocial determinants influencing exercise behavior in PD patients. Accumulating evidence suggests that psychosocial factors play an important role on exercise engagement, such as self-efficacy, social support, depression, apathy, perceived control, coping, and psychological resilience. Self-efficacy emerges as the most robust predictor across studies. Individuals who reported high exercise self-efficacy were more likely to engage in regular exercise regardless of disease severity. Social support from family, peers, and healthcare professionals could enhance the motivation, accountability, and adherence of exercise behavior. Depression and apathy significantly undermine exercise participation. Higher perceived control, active coping, and higher psychological resilience are associated with greater exercise engagement. This mini-review critically evaluated the evidence for these key psychosocial determinants in PD patients and proposed a clinically-informed framework for integrating psychosocial strategies into exercise promotion.

## 1 Introduction

Parkinson’s disease (PD) is the second most common neurodegenerative disorder and the global burden of PD is expected to accelerate in the coming decades (1). PD is characterized by progressive loss of dopaminergic neurons in the substantia nigra, leading to the motor symptoms of tremor, rigidity, bradykinesia, and postural instability, as well as non-motor symptoms including cognitive impairment, depression, anxiety, apathy, and sleep disturbances (2).

While pharmacological treatments, particularly levodopa and dopamine agonists, effectively manage motor symptoms, they show limited efficacy for non-motor symptoms and cannot halt or reverse disease progression (3). Moreover, long-term medication use is associated with complications including motor fluctuations and dyskinesias (4). Surgical interventions such as deep brain stimulation are invasive, expensive, and not universally available (5). This therapeutic gap has intensified interest in non-pharmacological approaches, such as physical exercise (6).

A substantial and growing body of evidence demonstrates that regular physical exercise confers multidimensional benefits for PD patients. For example, exercise interventions improve gait velocity, balance, functional mobility, and cardiovascular fitness (7). Importantly, exercise also enhances cognitive function and reduces depressive symptoms (8, 9). Emerging evidence from animal models and human studies suggests that exercise may exert neuroprotective effects by increasing brain-derived neurotrophic factor (BDNF) expression, reducing neuroinflammation, promoting neuroplasticity, and enhancing mitochondrial function (10, 11).

Unfortunately, population-level physical activity among PD patients remains strikingly low. Only approximately 27% of PD patients meet recommended guidelines of at least 150 min of moderate-intensity aerobic activity per week (12). Physical activity levels, in terms of both total volume and intensity, are significantly lower than age-matched healthy controls even in early disease stages. Dropout rates from structured exercise programs reach 50% within the first 6 months, and long-term maintenance of exercise behavior is rarely achieved (13). This research-to-practice gap represents a critical public health challenge that demands urgent attention.

Understanding why PD patients unsuccessfully engage in regular exercise is essential for developing effective interventions. While existing reviews have examined psychosocial factors in PD, they have predominantly focused on either broad quality-of-life outcomes (14) or general physical activity correlates without providing a clinically actionable framework (15). This mini-review distinguishes itself from previous work by: (1) critically synthesizing evidence specifically focused on the most robust psychosocial predictors of exercise behavior; (2) identifying key methodological gaps and controversies that limit current understanding; and (3) proposing a practical, theory-informed framework for integrating these determinants into multidisciplinary PD care. This review offers a unique contribution that bridges the gap between psychosocial research and clinical practice.

We searched in the PubMed, Web of Science, Wiley Online Library, and Springer in 2026 using combinations of keywords including “Parkinson’s disease,” “exercise behavior,” and “psychosocial factors.” The inclusion criteria were (1) publication date between 1996 and 2026; and (2) publication in a scholarly peer-reviewed journal. Totally, 624 articles were searched. Exclusion criteria were (1) unsuitable studies (e.g., irrelevant to this mini-review); (2) duplicate studies; and (3) no clear assessments/descriptions of psychosocial factors and exercise behavior. Ultimately, 35 articles were included (see Supplementary Table S1 for more details).

## 2 Key psychosocial determinants

According to relevant literature, several key psychosocial factors on exercise engagement in PD patients have been summarized.

### 2.1 Self-efficacy

Self-efficacy, defined as the belief in one’s capability to organize and execute courses of action required to attain specific goals, is the most consistently supported psychosocial predictor of exercise behavior across chronic illness populations (16, 17). A previous study found that self-efficacy was the best predictor of physical resistance and whether exercise was maintained up to 12 months post-intervention (18). The multicenter study of 260 PD patients by Ellis et al. (16) provided the most compelling evidence for the role of self-efficacy. Using the Self-Efficacy for Exercise scale, this study found that patients with high self-efficacy were more likely to engage in regular exercise than those with low self-efficacy, when disease severity, depressive symptoms, and demographic factors were controlled.

Bandura (17) proposed that self-efficacy influences behavior through four principal mechanisms: (1) choice of activities and environments (individuals with high self-efficacy choose more challenging tasks); (2) effort expenditure and persistence (high self-efficacy leads to greater effort and persistence in the face of obstacles); (3) thought patterns and emotional reactions (high self-efficacy reduces anxiety and catastrophic thinking); and (4) resilience to setbacks (high self-efficacy facilitates recovery from failures).

In PD patients, low self-efficacy may manifest as avoidance of physical activity due to fear of falling, beliefs that exercise will worsen symptoms, or lack of confidence in the ability to manage exercise-related discomfort (19). These beliefs are often reinforced by negative experiences, such as falls during walking or fatigue after activity, creating a self-perpetuating cycle of inactivity and functional decline. Breaking this cycle requires targeted interventions that provide performance accomplishments (e.g., progressively challenging exercise), vicarious experience (e.g., peer modeling), verbal persuasion (e.g., encouragement from healthcare providers), and management of physiological arousal (e.g., relaxation techniques) (20).

### 2.2 Social support

Social support is a consistently identified determinant of exercise behavior across chronic illness populations (21, 22). Social support encompasses various dimensions, including emotional support (e.g., encouragement, empathy), informational support (e.g., advice, guidance), instrumental support (e.g., practical assistance), and companionship (e.g., exercising together) (23). Support can come from multiple sources, including family members, friends, healthcare professionals, and exercise partners.

The mechanisms linking social support to exercise behavior are multifaceted. Social support may enhance self-efficacy through verbal persuasion and modeling. For example, seeing others successfully exercise provides vicarious experience that boosts confidence (24). Social support also reduces stress, which can undermine motivation and energy for exercise (25). Additionally, exercising with others provides accountability and creates a sense of obligation that increases adherence (26).

Among 672 PD patients surveyed in Netherlands, environmental factors including encouragement from partners were rated as influential motivators (27). A qualitative study of PD patients identified “exercising with a partner” as a key facilitator, with participants reporting greater motivation and enjoyment in social exercise contexts (28). Another study found that social support from significant others predicted exercise adherence, particularly when support was specific and encouraging rather than controlling or critical (29).

### 2.3 Depression

Depression is prevalent in PD patients and is associated with reduced physical activity (30). Depression is characterized by persistent low mood, anhedonia (loss of interest or pleasure), fatigue, sleep disturbances, and reduced motivation (31).

In an online survey of 672 PD patients, depression was negatively associated with current motivation to exercise (27). A multicenter study of 260 PD patients showed that exercisers had significantly lower Geriatric Depression Scale scores than nonexercisers (16). The relationship between depression and exercise is likely bidirectional with either virtuous or vicious cycles. Depression reduces motivation and energy for exercise, while exercise improves mood through neurochemical mechanisms (e.g., increased serotonin and dopamine) and psychosocial mechanisms (e.g., enhanced self-efficacy, social connection) (32).

### 2.4 Apathy

Apathy, independent of depressive mood, defined as diminished goal-directed behavior, may be particularly detrimental for exercise engagement (33), because it directly undermines the motivation needed to initiate and sustain behavior (34).

Apathy may be an even stronger predictor of exercise behavior than depression. A previous study (35) pointed out that apathy was associated with reduced engagement in health-promoting behaviors independently of depression, suggesting that apathy represents a distinct motivational deficit that cannot be fully captured by depression measures. Thus, strategies that address depression may not be sufficient for individuals with primary apathy, who may require behavioral activation or other approaches specifically targeting apathy.

### 2.5 Perceived control and coping

Perceived control, defined as the belief that one can influence outcomes through one’s actions, is a powerful determinant of health behaviors (36). In PD, higher perceived control is associated with better psychological adjustment, reduced depression, and greater engagement in health-promoting behaviors including exercise (37).

A study of PD patients found that perceived control mediated the relationship between stigma and well-being, suggesting that enhancing control beliefs may buffer the negative effects of social adversity (38). Similarly, perceived control has been shown to moderate the intention-behavior relationship, indicating that individuals who believe they have control over their exercise behavior are more likely to translate intentions into action (39).

Coping strategies also influence exercise behavior. Active coping (e.g., problem-solving, seeking information) is associated with greater exercise engagement, while avoidant coping (e.g., denial, behavioral disengagement) predicts lower activity levels (40). Individuals who actively problem-solve barriers are more likely to maintain exercise routines than those who passively accept obstacles as insurmountable. Adaptive coping skills could help individuals translate intentions into action by automating behavioral responses to anticipated barriers (41).

### 2.6 Psychological resilience

Resilience refers to the ability to adapt positively to adversity, which emerges as an important determinant of health outcomes across neurodegenerative diseases (42). In PD patients, higher resilience is associated with less depression, anxiety, and apathy, as well as better quality of life (43). A cross-sectional study of 138 PD patients found that resilience contributes to better chronic disease adjustment, suggesting that resilience plays critical role in PD adjustment (44).

The mechanisms linking resilience to exercise behavior are not yet fully understood. Resilience may enhance exercise engagement by promoting adaptive coping, maintaining positive affect, and sustaining motivation in the face of obstacles (45). Conversely, exercise may enhance resilience through neurobiological mechanisms (e.g., increased BDNF, reduced inflammation) and psychological mechanisms (e.g., reducing negative emotions, fulfilling individual needs) (46). This reciprocal relationship suggests a positive feedback loop in which exercise builds resilience, and resilience facilitates continued exercise.

Evidence for the resilience-exercise relationship in PD is limited but growing. Qualitative studies have identified resilience as a key factor in successful adaptation to PD. To be specific, resilient individuals are more likely to engage in exercise and other health-promoting behaviors (47).

### 2.7 Disease-related and demographic factors

Disease severity and physical impairments are also predictors of exercise behavior.

In a cross-sectional study of 260 PD patients, disease severity was significantly associated with exercise status in univariate analysis (16). The willingness to participate in activities will decrease with disease progression.

Demographic factors show mixed associations with exercise behavior. Higher education is consistently associated with greater exercise engagement, which reflects greater health literacy and financial resources (16, 48). Age showed a small positive association in some studies, possibly because older individuals are more aware of age-related health risks or have more time for exercise (16). But another study showed that current motivation levels to exercise are associated with lower age (27). The effects of sex on exercise behavior are also inconsistent. Some studies found men more likely to exercise (49), but some researches found there was no difference between men and women in exercise (16, 27).

Additionally, outcome expectations, fatigue, and health literacy are all well-established determinants (48). Outcome expectations, defined as the belief that whether exercise will yield meaningful benefits, serve as a major barrier to initiating exercise. Positive expectations reinforced by education and personal experience can strongly motivate exercise engagement. Fatigue directly undermines exercise participation by creating a perceived physical and psychological obstacle, though some evidence suggests that regular exercise may eventually alleviate it. Health literacy, reflected in patients’ understanding of exercise’s role in disease management, determines whether they can integrate physical activity into daily life.

Briefly (Table 1), in PD patients, self-efficacy is the strongest psychosocial predictor of exercise behavior (16). Social support enhances adherence through encouragement, modeling, and accountability (24–26). Depression and apathy strongly undermine physical activity (30, 33). Perceived control and active coping facilitate translation of intentions into action (37, 40). Psychological resilience appears to both enable and result from exercise (45, 46).

**Table 1:** Summary of reviewed psychosocial factors.

Factors	Key findings	Mechanisms of exercise
Self-efficacy	Individuals with high self-efficacy are more likely to exercise (16)	① Choice of challenging activities; ② greater effort and persistence; ③ reduced anxiety and catastrophic thinking; ④ enhanced resilience to setbacks (17)
Social support	Individuals with high social support are more likely to exercise (21, 22)	① Enhances self-efficacy through verbal persuasion and modeling (24); ② buffers stress (25); ③ provides accountability and creates sense of obligation that increases adherence (26)
Depression	Depression is associated with reduced physical activity (30)	Depression reduces motivation and energy for exercise (32)
Apathy	Apathy may be particularly detrimental for exercise engagement (33)	Apathy directly undermines initiation and maintenance of goal-directed behavior (34)
Perceived control and coping	Higher perceived control and active coping are associated with greater exercise engagement (37, 40)	① Perceived control buffers negative effects of social adversity (38); ② enhances translation of intentions into action (39); ③ active coping automates behavioral responses to anticipated barriers (41)
Psychological resilience	Resilient individuals are more likely to engage in exercise and other health-promoting behaviors (47)	① Resilience promotes adaptive coping, positive affect, and sustained motivation despite obstacles (45); ② exercise enhances resilience via neurobiological and psychological mechanisms (46)
Disease-related and demographic factors	Disease related and demographic factors are also predictors (16)	① Movements become difficult with disease progression (16); ② education likely reflects greater health literacy and financial resources (16)

## 3 Research gaps and controversies

Although evidence is growing, gaps and controversies remain in the literature on psychosocial determinants of exercise behavior in PD.

### 3.1 Methodological heterogeneity and sample selection bias

Substantial methodological heterogeneity, including varied definitions of “regular exercise” and diverse measures, limits comparability across studies. For example, some studies define exercisers as those who exercise at least three times per week for 20 min (16), but others use broader definitions. The same psychosocial factor is often measured using different instruments across studies. For instance, resilience has been assessed by the Connor-Davidson Resilience Scale, the Resilience Scale for Adults, and the Resilience Scale-15 in different studies (42). Study samples are predominantly white, highly educated, and with mild-to-moderate disease severity (16, 27). This homogeneity substantially constrains the generalizability of findings. Tailored interventions require understanding which factors are most influential for specific subgroups (50). For example, collectivist cultures may place greater emphasis on social support, while individualist cultures may prioritize self-efficacy.

### 3.2 Limited longitudinal and causal evidence

Although cross-sectional studies can identify associations between psychosocial factors and exercise behavior, they cannot establish directionality or causality. For example, the depression-exercise association is likely bidirectional, with each influencing the other over time (32). However, the scarcity of longitudinal studies in PD populations means that the temporal precedence of these relationships remains largely unknown. The few existing longitudinal studies suggest that self-efficacy predicts exercise maintenance up to 12 months (18), but these findings require replication in larger and more diverse samples. Furthermore, no large-scale randomized controlled trial has specifically targeted self-efficacy, social support, perceived control, or resilience as the primary factor for improving exercise adherence in PD. While observational studies consistently identify these factors as correlates, the absence of experimental evidence leaves the causal efficacy of these determinants unproven.

### 3.3 Lack of integrated theoretical models and mechanistic understanding

Complex theoretical models using structural equation modeling or path analysis to simultaneously test multiple pathways and mediators are rarely employed. For example, the pathways between resilience and exercise behavior are not well established with failing to distinguish between direct and indirect effects mediated by self-efficacy or other factors (46). A previous study tested a model of explicit memory, perception of social resources, depression, and quality of life, providing a methodological template (51). While studies have identified neurobiological mechanisms through which exercise improves brain function (10), the mechanisms of the effect of psychosocial factors on the exercise-brain relationship are poorly understood. For instance, it is unclear whether high self-efficacy enhances the BDNF response to exercise or whether depression attenuates it.

## 4 Clinical implications and recommendations

Despite the gaps and controversies noted above, the existing evidence provides a compelling rationale for integrating psychosocial approaches into exercise promotion for PD patients. Several practical strategies for enhancing exercise engagement in PD patients were suggested, ideally led by multidisciplinary teams that include clinicians, occupational therapists, psychologists, and nurses, depending on the specific needs of the patient.

### 4.1 Addressing self-efficacy

Self-efficacy is modifiable through structured interventions. For individuals with low self-efficacy, strategies include: (1) setting specific, measurable, time-bound, achievable, and progressively challenging goals (e.g., “walk for 10 minutes three times this week”) that provide experiences of success (52); (2) using peer models and success stories to demonstrate that exercise is possible despite PD; (3) providing encouragement and positive feedback, emphasizing effort and improvement rather than outcomes; and (4) teaching relaxation techniques and symptom management strategies that help individuals tolerate exercise-related discomfort and interpret physiological signals (e.g., increased heart rate, fatigue) as normal rather than threatening (17).

### 4.2 Enhancing social support

Evidence suggests that group exercise may improve adherence more than home-based programs for some individuals (53). To enhance social support, healthcare professionals should: (1) recommending exercise classes or support groups that provide opportunities for social connection and shared experience (54); (2) connecting patients with peer mentors who have successfully maintained exercise routines; (3) encouraging family members to express confidence in the patients’ abilities and offer practical assistance when needed; and (4) using technology to facilitate social support, including online exercise groups, social media communities, and videoconference-based exercise classes (55).

### 4.3 Screening and treating depression/apathy

Screening and treating depression and apathy may be essential prerequisites for successful exercise engagement (56). Routine screening for depression and apathy should be integrated into PD care with brief screening tools such as the Geriatric Depression Scale (57). When depression or apathy is identified: (1) applying pharmacotherapy (e.g., antidepressants) and/or psychotherapy (e.g., cognitive-behavioral therapy) for depression (58); (2) exploring behavioral activation techniques for apathy that emphasizes the link between activity and mood (59); and (3) adjusting exercise programs to maximize enjoyment for individuals with mood disturbances, including shorter sessions, lower intensity, more frequent breaks, and incorporation of preferred activities.

### 4.4 Fostering perceived control and coping

Perceived control can be enhanced through interventions that provide choice, mastery experiences, and self-monitoring opportunities (60). Rather than prescribing a “one-size-fits-all” regimen, healthcare professionals should: (1) helping patients identify personally meaningful exercise goals that align with their values and life priorities; (2) offering a menu of exercise options (e.g., Tai Chi, yoga, walking, cycling, resistance training) for patients to find enjoyable and appropriate options (48); (3) encouraging self-monitoring and self-adjustment based on daily energy levels and symptoms (61); and (4) developing exercise plans and coping plans that specify when, where, and how exercise will be performed, as well as how to overcome anticipated obstacles such as fatigue, pain, and motivation fluctuations (41).

### 4.5 Enhancing psychological resilience

Enhancing resilience may indirectly promote exercise engagement. Key approaches include: (1) selecting adapted and tailored interventions such as cognitive-behavioral therapy, mindfulness-based stress reduction, and positive psychology approaches (62); (2) increasing awareness of mental health needs including reducing stigma and proactively offering psychological support; and (3) scheduling regular debriefings after stressful events or shifts to review what worked and what needs improvement (63).

## 5 Limitations

This mini-review has several limitations. First, this study used narrative synthesis approach and lacked of a systematic quality assessment or a meta-analytic model, which limits the strength of conclusions. Second, the majority of cited evidence is cross-sectional, preventing causal inference about the direction of relationships between psychosocial factors and exercise behavior. Third, this study is heavily reliant on a small number of large-scale studies and these findings may not be generalizable to all PD populations, particularly those in non-Western countries or with more advanced disease stage. Forth, it should be explicitly acknowledged that this mini-review may not be comprehensive. The scope of the literature covered is inherently constrained by the search strategies, databases, and time frames, thus relevant studies may have been inadvertently omitted.

## 6 Conclusion

Physical exercise is one of the most promising non-pharmacological interventions for PD patients, yet adherence remains a significant challenge. Psychosocial determinants, including self-efficacy, social support, depression, apathy, perceived control, coping, and resilience, play a crucial role in exercise engagement. Clinical implications emphasize person-centered approaches to enhance exercise adherence and optimize outcomes for PD patients, contributing to improve quality of life and reduce disability in this growing population.
