# Supplementary material for: Psychosocial determinants of exercise behavior in Parkinson’s disease: a mini-review
Source: Front Public Health. 2026 Sep 3;14:1942675. doi: 10.3389/fpubh.2026.1942675 (PMC13581726; doi:10.3389/fpubh.2026.1942675)
Supplement: Supplementary file 1 [file Data_Sheet_1.PDF]

Table S1: The main characteristics of the selected articles included in this mini-review.

Study	Psychosocial Factor(s)	Measurement Tool(s) / Method
Ellis et al., 2011 [16]	Self-efficacy; Depression; Disease severity; Demographic factors	Self-Efficacy for Exercise (SEE) scale; Geriatric Depression Scale (GDS); Unified Parkinson's Disease Rating Scale (UPDRS)
Bandura, 1997 [17]	Self-efficacy	Conceptual review
Neupert et al., 2009 [18]	Self-efficacy	Exercise Self-Efficacy scale
Afshari et al., 2017 [19]	Self-efficacy	Qualitative interviews
Marks et al., 2005 [20]	Self-efficacy	Review synthesis
Lindsay Smith et al., 2017 [21]	Social support	Systematic review; various self-report social support scales
Rackow et al., 2015 [22]	Social support	Berlin Social Support Scales (BSSS)
Thoits, 2011 [23]	Social support	Conceptual review
Anderson et al., 2010 [24]	Social support; Self-efficacy; Outcome expectations	Social Support for Exercise scale; Self-Efficacy scale; Outcome Expectations scale
Cohen & Janicki-Deverts, 2009 [25]	Social support	Conceptual review
King, 2001 [26]	Social support	Review
Cools et al., 2025 [27]	Depression; Social support; Demographic	Geriatric Depression Scale (GDS); Online survey

Study	Psychosocial Factor(s)	Measurement Tool(s) / Method
	factors	
Flynn et al., 2022 [28]	Social support	Qualitative semi-structured interviews
Ravene & Schneider, 2009 [29]	Social support; Perceived control	Social Support for Physical Activity scale; Perceived Control questionnaire
Marsh, 2013 [30]	Depression	Review; various depression scales
Prange et al., 2022 [31]	Depression	Review
Schuch et al., 2018 [32]	Depression	Meta-analysis of prospective cohort studies; various depression scales
den Brok et al., 2015 [33]	Apathy	Systematic review and meta-analysis; various apathy scales
Simpson et al., 2015 [34]	Apathy	Qualitative interviews
Harrison et al., 2023 [35]	Apathy	Apathy Evaluation Scale (AES)
Salehi et al., 2016 [36]	Perceived control	Perceived Control questionnaire
Eccles & Simpson, 2011 [37]	Perceived control	Systematic review; various perceived control measures
Verity et al., 2020 [38]	Perceived control;	Perceived Control scale
Hagger et al., 2022 [39]	Perceived behavioral control	Meta-analysis
Serier et al., 2018 [40]	Coping	Coping Strategies Inventory (CSI)

Study	Psychosocial Factor(s)	Measurement Tool(s) / Method
Gollwitzer, 1999 [41]	Coping	Conceptual review
Ovaska-Stafford et al., 2021 [42]	Psychological resilience	Systematic review; various resilience scales
Robottom et al., 2012 [43]	Psychological resilience	Connor-Davidson Resilience Scale (CD-RISC)
Shamaskin-Garroway et al., 2016 [44]	Psychological resilience	Resilience Scale for Adults (RSA)
Silverman et al., 2017 [45]	Resilience; Coping	Qualitative interviews
Jiang et al., 2025 [46]	Resilience	Systematic review; various resilience measures
McCabe & O'Connor, 2012 [47]	Resilience	Resilience scale
Schootemeijer et al., 2020 [48]	Demographic factors; Health literacy	Survey; Health Literacy measures
Cumming et al., 2008 [49]	Demographic factors	Demographic questionnaire
Schootemeijer et al., 2020 [50]	Outcome expectations; Fatigue; Health literacy	Survey; Health Literacy Scale
